# Evaluation of a Salutogenetic Concept for Inpatient Psychosomatic Treatment

**DOI:** 10.1155/2013/735731

**Published:** 2013-09-16

**Authors:** Thilo Hinterberger, Jochen Auer, Stephanie Schmidt, Thomas Loew

**Affiliations:** ^1^Research Section of Applied Consciousness Sciences, Department of Psychosomatic Medicine, University Medical Center Regensburg, 93047 Regensburg, Germany; ^2^Institute of Psychology, Leopold Franzens University Innsbruck, 6020 Innsbruck, Austria; ^3^Department of Psychosomatic Medicine, University Medical Center Regensburg, 93053 Regensburg, Germany

## Abstract

The increase of psychosomatic disorders due to cultural changes requires enhanced therapeutic models. This study investigated a salutogenetic treatment concept for inpatient psychosomatic treatment, based on data from more than 11000 patients of a psychosomatic clinic in Germany. The clinic aims at supporting patients' health improvement by fostering values such as humanity, community, and mindfulness. Most of patients found these values realized in the clinical environment. Self-assessment questionnaires addressing physical and mental health as well as symptom ratings were available for analysis of pre-post-treatment effects and long-term stability using one-year follow-up data, as well as for a comparison with other clinics. With respect to different diagnoses, symptoms improved in self-ratings with average effect sizes between 0.60 and 0.98. About 80% of positive changes could be sustained as determined in a 1-year follow-up survey. Patients with a lower concordance with the values of the clinic showed less health improvement. Compared to 14 other German psychosomatic clinics, the investigated treatment concept resulted in slightly higher decrease in symptoms (e.g., depression scale) and a higher self-rated mental and physical improvement in health. The data suggest that a successfully implemented salutogenetic clinical treatment concept not only has positive influence on treatment effects but also provides long-term stability.

## 1. Introduction

Since 1948, the World Health Organization defines health as “a state of complete physical, mental, and social well-being and not merely the absence of disease or infirmity” [1]. This definition suggests a dichotomous categorization into states of absolute health and not health or disease, which cannot be adequately addressed by health care systems and insurances. Our medical system is set up for the treatment of specific diseases, characterized by the presentation of symptoms or reports, which can be interpreted as an aberration of a physiological equilibrium or norm and which can be attributed to certain internal or external impairments [2]. The diagnosis of diseases is thus strongly influenced by social, temporal, and local factors. This becomes obvious when looking at internationally used diagnostic tools such as the International Classification of Diseases (ICD-10). For example, hysteria, a once-common medical diagnosis [3], is no longer recognized as individual illness and not part of the current version of the International Classification of Diseases (ICD-10) [4]. In Europe, an Indian Yogi in his advanced, enduring state of meditation, would be diagnosed with catatonic schizophrenia according to the ICD-10 F20.2 [4]. An increasingly diagnosed group of symptoms known as “burnout” syndrome is not recognized as disease in the ICD-10. These examples illustrate that the definition of disease is dependent on social conditions and norms and thus, a tool of health care policy.

Over the last century, a major shift in the distribution of disease types has occurred. While in the last century the health system had to cope predominantly with infectious diseases [5], today psychosomatic diseases are increasing rapidly [6] which makes it necessary to address the question how humans can stay healthy in a psychologically challenging environment. Based on the observation that some people seem resistant to stressful experiences [7], researchers investigated health promoting behaviors and found them critical for health improvement and maintenance [8, 9]. These health supporting behaviors include the promotion of mindfulness [10], spirituality [11], social support [12–14], and a sense of coherence (SOC) [15]. 

The SOC concept was introduced by Antonovsky and encompasses comprehensibility, manageability, and meaningfulness [7, 16]. Meaningfulness refers to the belief that one's life or at least some aspects of life are interesting, have a meaning, are worth spending energy on, and are considered as an enjoyable challenge. Comprehensibility is the feeling that things in life happen in an organized and predictable way. Manageability is the belief that life events are within one's control [17]. Individuals with a greater SOC also tend to make healthier lifestyle choices and have a higher intake of fruit and vegetables, greater physical activity, and decreased likelihood of smoking [18]. 

Schumacher et al. [19] examined Antonovsky's Sense of Coherence Scale on healthy Germans, demonstrating significant correlations between comprehensibility, manageability, and meaningfulness and proposed a global factor. There were also significant correlations between SOC and age (age-correlated decline of SOC scores), sex (lower SOC scores by women), and psychological/physiological well-being (higher SOC was related to less somatoform symptoms).

Höfer and Straus [20], Bafiti [21], and Simonsson et al. [22] showed in their studies high inverse correlations between SOC and psychosomatic symptoms, underlining the positive effect of SOC on health. Tselebis et al. [23] reported a significant inverse correlation between SOC and both burnout and depression. Further research confirmed the relatedness of higher SOC with less psychosomatic and physiological symptoms [24–27]. Finally, a review study analyzing 458 scientific publications and 13 doctoral theses on this topic reported a strong correlation between SOC and perceived health, especially mental health [28].

Antonovsky [16] views meaningfulness as the essential motivational component of SOC, having the greatest influence on health. Without the experience of meaningfulness, the overall feeling of coherence is diminished, despite a possible high intensity of the other two components. In their recent study with 232 participants, Parker et al. [29] confirmed that meaningfulness is more important than comprehensibility and manageability. Another important factor for health is social support, which is positively related to SOC [14]. 

Another extended health model is the biopsychosocial model (BPS) proposed by Engel. The BPS integrates biological information from the patient's body, psychological aspects such as negative thinking or lack of self-control, and social factors such as socioeconomic status and religion to assess the causes of illness, aiming to bridge the mind-body connection [30, 31]. However, as pointed out by many authors, this model is a thought construct without elaborating on the details [32–34]; also, the model does not include the concept of a personal sense of meaning and spirituality, which are crucial to health. Increased spirituality has been associated with the prevention of health risk behavior [35] as well as a greater sense of control and other effects on health [36]. 

Another construct that can be included into the concept of salutogenesis is mindfulness. The most common psychological definitions of mindfulness mainly describe the construct by emphasizing two points: firstly, full attention to the present moment on immediate experience. Secondly, this awareness should be experienced in a state of equanimity, whatever sensation arises, without judgment, elaboration and reaction [37, 38]. This state of awareness can be trained through mindfulness exercises, and if applied in everyday life, a trait can develop. Furthermore, mindfulness training can increase spirituality [39, 40] and general SOC [41]. Based on prior research, mindfulness can be regarded as a health supporter [42–44]. We would like to broaden the purpose of mindfulness exercises (in the sense of old religious traditions, see, e.g., [41]) to the effect that they can also open up access to personal spirituality and finding a personal sense of meaning.

In the present study we intend to show the treatment effects of a psychosomatic clinic which aims to implement the above-mentioned salutogenetic factors. We will investigate how these factors and values are realized in the treatment, and analyze the influence of personal concordance on treatment success.

*Concept of Psychosomatic Treatment in Heiligenfeld*. Heiligenfeld (HF) is a psychosomatic clinic which was established on the theory of SOC. The overall concept of the clinic is designed to support salutogenetic factors. HF is embedding these values in the organization of the clinic and in the psychotherapeutic community. In addition to the essential component of meaningfulness in the SOC, HF extends this component with a transcendent meaning of life. HF considers itself not merely as a hospital but rather as a place of health and humanity that aims at substantiating basic humanistic values and ways of finding meaning, as covered below. The central idea is to promote holistic intellectual development, a self-determined and autonomous life, the meaningfulness of personal and entrepreneurial action in a place of recovery, and humanity. These values are represented and lived by the team of therapists and all employees (including the kitchen and cleaning staff). Awareness and self-realization, mindfulness, personal responsibility and self-determination, humanity and human dignity, care and love for the fellow human being, authenticity, and sincerity qualify every staff member and are intended to be lived when working together with the patients. Every employee sees himself/herself as part of the whole process of therapy, and their behavior is thought to inspire and enable patients to start the search for self-realization and meaning. Hence, HF operates under the guideline of Jung [45]: “you cannot tear the human being into two pieces, to delegate one part to the physician and one part to the theologian.” The pursuit of finding meaning in life which can lead to an increase in SOC and spirituality is a consistent part of a patients' daily routine—a routine that accompanies him or her until the end of hospitalization. Mindfulness plays a significant role, not only as therapeutic intervention but also as a central method to increase (self-) awareness, to connect with one's spirituality and to find meaning. The medical and psychotherapeutic treatment is based on a holistic concept and combined with body-related psychotherapy, expressive arts therapy, reality-oriented social therapy, relaxation techniques, and meditation.

 A positive correlation between spirituality, for example, feeling centered, and therapeutic success in HF has been reported previously [46]. A study by Hawks et al. found that imagery, meditation, and group support activities may positively affect the sense of meaning and purpose in life, self-awareness and connectedness with self, others, and a larger reality [47]. 

*Therapeutic Community*. Basically, interwoven with the concept is the idea of the therapeutic community providing social support and granting an important healing frame for the patients. The therapeutic community allows the practical experience of meaningfulness and general SOC in daily life and also constitutes a crucial element for environment-based social-therapeutic work. 

This field comprises mainly the following:

 (i) general assembly (“Plenum”) on parting and for welcoming new patients (also open to visitors);
 (ii) assembly of all patients;
 (iii) therapeutic group for support and for improvement of communicative skills;
 (iv) sharing of community tasks and responsibilities;
 (v) psychoeducation in fields like health and disease, treatment concepts, and healthy nutrition;
 (vi) recreation offers for the weekend;
 (vii) involvement of patients in the improvement and complaint management.

*Short Description of Interventions*. The treatment program is tailored individually for every single patient. It connects group therapy, individual one-on-one therapy, psychoeducation, body oriented and creative treatments, movement and music therapy, relaxation techniques and meditation, and medical treatment. Therapeutic methods offered include different forms of meditation, Holotropic breath work, concentrative Breathwork and body therapy, voice therapy, rhythm therapy, dance therapy, water therapy, expressive painting, and therapeutic karate therapy with animal support.

The mental-spiritual aspect of human existence is addressed through various optional offers. Spirituality is actively supported by a systematical introduction and daily exercises of mindfulness meditation, daily changing meditation sessions (guided, silent, moving, contemplative observing the sense of words and useful phrases or basic questions of human life and death, and visualization), the option to practice in a separate meditation room all day, daily self-reflection, autogenic training, and other relaxation techniques.

In this context it is important to mention the experimentally confirmed motivational concordance theory by Hyland et al. [48–50], proposing that the extent to which therapeutic rituals fit the individual's belief systems or personal goals determines the treatment effect. 

*Goals of Our Study*. Our study is based on the investigation of how salutogenetic factors and values are realized in HF. Although the assessment scheme did not measure the above-mentioned salutogenetic factors through specific questionnaires, several salutogenetic components could be tested with a set of questionnaires offered by the clinic.

We will assess treatment effects, also dependent on the respective diagnosis or treatment path, and also analyze the effect of personal concordance on treatment success and sustainability of effects. Finally we will compare treatment effects with other clinics.

## 2. Methods

### 2.1. Design

**Figure 1:**
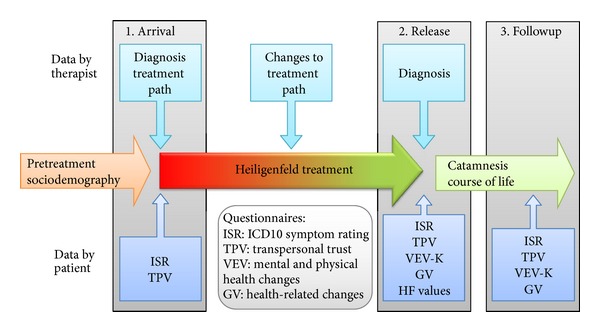
The data pool describing a patient in the therapeutic setting of Heiligenfeld consists of the patient's history and demography, the psychological and somatic diagnostic data provided by the therapist, and a number of questionnaires that were distributed to the patients. Questionnaires were completed by the patients before, directly after, and one year after treatment.

Patients of the HF clinics were asked to complete several self-assessment questionnaires and surveys at their arrival (before treatment) and at their release (after treatment) within a few days. The reception survey also asked for the sociodemographic situation. For a followup, some of the patients received a selection of questionnaires again one year after treatment (see Figure 1).

Patients entering the clinic were initially diagnosed by a medical doctor as well as by a psychologist in accordance with ICD-10. Based on the main psychological diagnosis, they were assigned to one of 13 treatment paths (for details see below). A treatment path is defined by the compilation of therapeutic interventions and groups depending on the main diagnosis for a more specific treatment of various diagnoses.

### 2.2. Materials

#### 2.2.1. Questionnaires

Our analysis is based on several self-assessment questionnaires, which are used to measure symptoms, work-related coping behavior, personal beliefs, and habits. The questionnaires were in German, the native language of the participants, and are presented below.

*ICD-10 Symptom Rating (ISR)*. The ISR is an ICD-10 based symptom rating inventory. The ISR is a license-free symptom-rating inventory that roughly corresponds to the internationally well-known SCL90 (Symptom Checklist 90) [51]. In a comparative study, the total scales of ISR and SCL-90 had a correlation of *r* = 0.833, but the intercorrelations were lower (*r* = 0.32) in the ISR than in the SCL-90 (*r* = 0.65) [52]. The ISR consists of 29 items in a scale of 5 options leading to the factors depression, anxiety, obsession, somatic symptoms, eating disorders, and additional supplementary factors addressing suicide, problems with sleep, memory, sexuality, or traumatic experiences [51, 53, 54]. Cronbach's alpha is 0.92 for the total score and between 0.78 and 0.86 for the syndrome scales [55].

*Transpersonal Trust (TPV)*. The TPV (from German “Transpersonales Vertrauen”) was designed by Bantelmann [56] and Belschner [57] to assess transpersonal confidence, faith, and beliefs. It is part of a larger inventory set, the FIG-50 (Questionnaire Integral Health “Fragebogen Integrale Gesundheit 50”). In the TPV, 10 questions measure an individual's spirituality in a scale of 4 options. Its validity has been confirmed [58], and it has an internal consistency (Cronbach's alpha) of 0.92 as referenced in [57, 59].

*Health-Related Total Change (GV)*. The GV (from German “Gesamtveränderung”) assesses global changes in mental and physical states. It consists of 11 questions addressing the aspects of somatic and mental improvement and coping abilities. The scale had 7 options, defined as −2 = “essential deterioration,”  −1 = “some deterioration,”  0 = “unchanged,” 1 = “some improvement,”  2 = “essential improvement,”  3 = “very much improvement,” and “was not my problem”. Thus, in the results a value of 1.20 would mean that patients had some improvement on the respective scale. This questionnaire is used across clinics by the Institute of Quality Development in Psychotherapy and Psychosomatics (IQP) within the basis documentation Psy-BaDo. Here, every item is analyzed separately and compared statistically across clinics. The questions ask for improvement in physical health, psychological health, self-esteem, social problems, private relationships, occupational abilities, motivation, disease-related comprehension, future outlook, well-being, and daily life requirements. Besides reporting a total score, we additionally report the following constructs: (1) *physical health* which addresses changes in physical well-being (item 1), (2) *mental health* that includes psychological health and emotional well-being (items 2 and 10), (3) *self-esteem* (item 3), and (4) *coping* including social relationships, occupational aspects, motivation, comprehension of disease, future orientation, and daily life requirements. The coping construct includes the ability to live a self-directed life, something that is explicitly supported and taught in the clinic. Thereby, important aspects of the SOC model as well as an extended view of salutogenesis may be assessed in the GV. For further details see the Supplementary Appendix in Supplementary Material available online at http://dx.doi.org/10.1155/2013/735731. 

*Assessment of Changes in Behavior and Experience (VEV-K)*. The VEV-K (from German “Vergleichsfragebogen zum Verhalten und Erleben,” “comparative questionnaire of behavior and experience”) is a short form of the VEV and contains 25 questions in a scale with 7 bipolar items from −3 to +3. It has confirmed validity and Cronbach's alpha of 0.9 [60–62]. Patients are asked to estimate their personal change after treatment in different areas. The questions can be associated with three factors: coping, social trust/future orientation, and resting/contentedness according to a factorial analysis performed on our data (we performed a principal component analysis on our VEV dataset (*n* = 8989). Via varimax rotation with Kaiser normalization we determined the three main factors). While the coping factors relate to self-management and meaningfulness, the social trust (items 19, 21–25) focuses on the inner confidence in social interactions. It should be noted that social support is connected with coping through the ability to manage social interactions. The resting/contentedness factor formed by items 1–5 represents a relaxed, peaceful, calm, and balanced state of being. This questionnaire addresses essential behavioral and perceptional properties which also form the concept of salutogenesis.

*Heiligenfeld Values (HFV)*. In a questionnaire of 14 items, patients were asked for their opinion on a scale of four options of how well the values of the clinic concept have been realized (see Supplementary Appendix). These values addressed HF to be a place of humanity, health, healing, love, wholeness, spiritual development, community, and mindfulness. Further, they should rate how well basic values of human being were realized, how well people are regarded, essentials were addressed, responsibility was lived, life was respected, and compassion was present. For the dataset used here, the value questionnaire showed a high reliability with Cronbach's alpha of 0.90. These values are thought to constitute a salutogenetic effect when realized in the clinical environment. Potentially, the SOC components could be found in the HFV in the sense that the aspect of meaningfulness, for example, can be created if essentials are addressed. Comprehensibility might be promoted by mindfulness or the presence of values of humanity. Manageability might be supported by the responsibility which is lived and the value of community.

#### 2.2.2. Quality Management and Benchmarking

The HF clinic is part of a benchmarking system, developed and managed by the Institute of Quality Development in Psychotherapy and Psychosomatics (IQP, Munich, Germany). The system is called BaDo (Psychosomatic Basis Documentation) [53] and allows for a comparison of treatment effects between the HF clinic and 14 other German hospitals in the version of 2011 which we used. For the comparison data were extracted from the evaluation report of the year 2011 that used BaDo version 4.1 [63]. This report compared 1541 patients of the HF clinic with a total of 7540 patients from other clinics. The ratio female/male was 67% in HF and 66% in other clinics. The average age was 47.6 years in HF and 44.3 years in the other clinics. While almost 70% of the HF patients had a higher educational level (German Abitur), this was the case for only 28% of patients in the other clinics.

### 2.3. Procedure

#### 2.3.1. Research Questions

The above-presented set of variables and their organization allowed for testing the following specific research questions.

 (Q1) How are the clinic-specific salutogenetic concept and the underlying values realized in the Heiligenfeld clinics?
 (Q2) How does the concordance between the ethical concept and its perception by the patient influence the patient's treatment success and salutogenesis?
 (Q3) What treatment effect sizes were achieved?
 (Q4) How are treatment effects dependent on the diagnosis or the treatment path?
 (Q5) How sustainable are treatment effects?
 (Q6) How do treatment effects compare to other clinics?

Table 1 illustrates the questionnaires we analyze for answering the above-mentioned research questions.

#### 2.3.2. Data Analysis

Raw data were received from HF. Data processing and the calculation of questionnaire factors and scales were performed according to the official manuals. Missing item data were replaced by the average of item values which contribute to a specific factor. This means that for a valid factor at least one item has to be answered, otherwise the patient is excluded from the data pool contributing to a specific factor. The calculation of effect sizes requires a normal distribution. Unfortunately, Lilliefors tests as well as Kolmogorov-Smirnov tests applied to the data of the factors of the ISR, TPV, VEV-K, and GV failed. However, a comparison between normal and actual distribution resulted in an acceptable fit (10% error) in the probability range between 10 and 90% for the ISR, TPV, and GV. The VEV-K showed an acceptable fit in the range between 15 and 80%, and the HFV only provided a good fit in the probability range of 25 to 75%, that is, values between 3.5 and 3.9 for the HFV total factor. The standard error bars in the VEV-K scores therefore have to be treated with caution. Cohen's d effect sizes of ISR factors and the TPV were calculated using the difference between pre- and posttreatment means (or follow-up mean) and divided by the pooled standard deviation.

## 3. Results and Discussion

### 3.1. Patient Population

Our study focuses on the inpatients in three clinics of the Heiligenfeld group which are all located in Bad Kissingen/Germany. Two clinical categories can be distinguished: one clinic for patients with a public health insurance (Fachklinik, 37.0%), and two clinics for patients with private health insurance (Parkklinik, 52.5% and Rosengartenklinik, 10.5%). 

After exclusion of patients who stayed less than 14 days in the hospital data from over 11,200 patients treated during 1/2007 to 1/2013 were available. This set consisted of 69% female and 31% male patients, aged between 17 and 74 years (average age 45.9 ± 9.9 years) who stayed in the hospital for 54.7 (±19.5) days on average. 57% of them had a higher educational level (university or similar). Patients with various psychosomatic disorders were included. The follow-up survey one year after treatment was effectively completed by about 1050 patients depending on the questionnaire.

**Figure 2:**
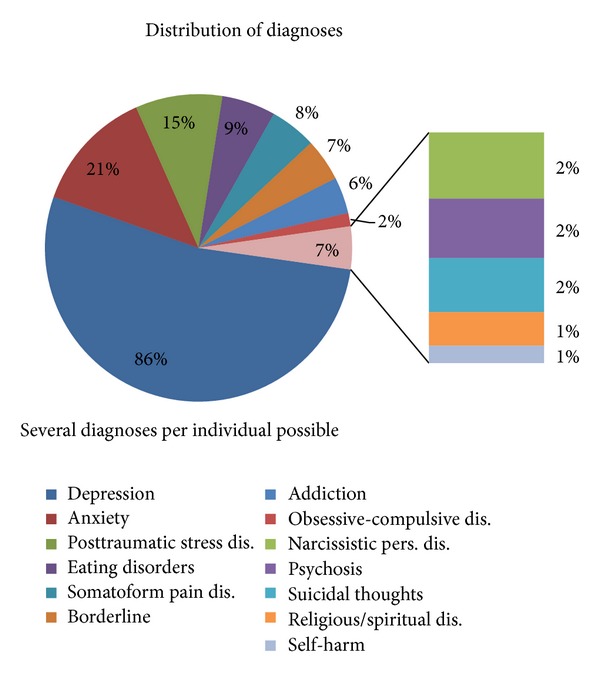
The distribution of patients into the various treatment paths is illustrated. Several diagnoses per individual are possible.

The most frequent treatment paths were depression (86.3%, *N* = 9468), anxiety disorders (21.2%, *N* = 2308), posttraumatic stress disorders (PTSD, 14.8%, *N* = 1631), eating disorders (9.8%, *N* = 1009), pain (7.9%, *N* = 871), Borderline (7.7%, *N* = 799), and addiction (6.9%, *N* = 680) (see Figure 2).

### 3.2. Realization of Values in Clinical Environment (Q1)

First, the patients' ratings are reported indicating how well they find the HF values realized in the clinic before in a second step (Q2) we explored how the concordance of patients with these values stands in connection with treatment success.  Table 2 illustrates how patients rated the clinical environment. Over 90% of patients voted “partly applies” or “fully applies” for each of the items according to Table 2. The best votes were given for the clinic being a place of humanity, community, and healing. Almost all patients partly or fully shared the opinion that basic values were realized, people are regarded in their dignity, and compassion is lived. As a result it can be stated that these values were perceived to be present to almost all patients during their stay. Unfortunately, due to the high agreement this questionnaire shows ceiling effects. Further statistics therefore suffers from distributional problems. Another weakness of the whole survey including this questionnaire was that it was personalized. This might bias the responses towards a higher conformity with the clinic.

### 3.3. Concordance Influences Treatment Success (Q2)

**Figure 3:**
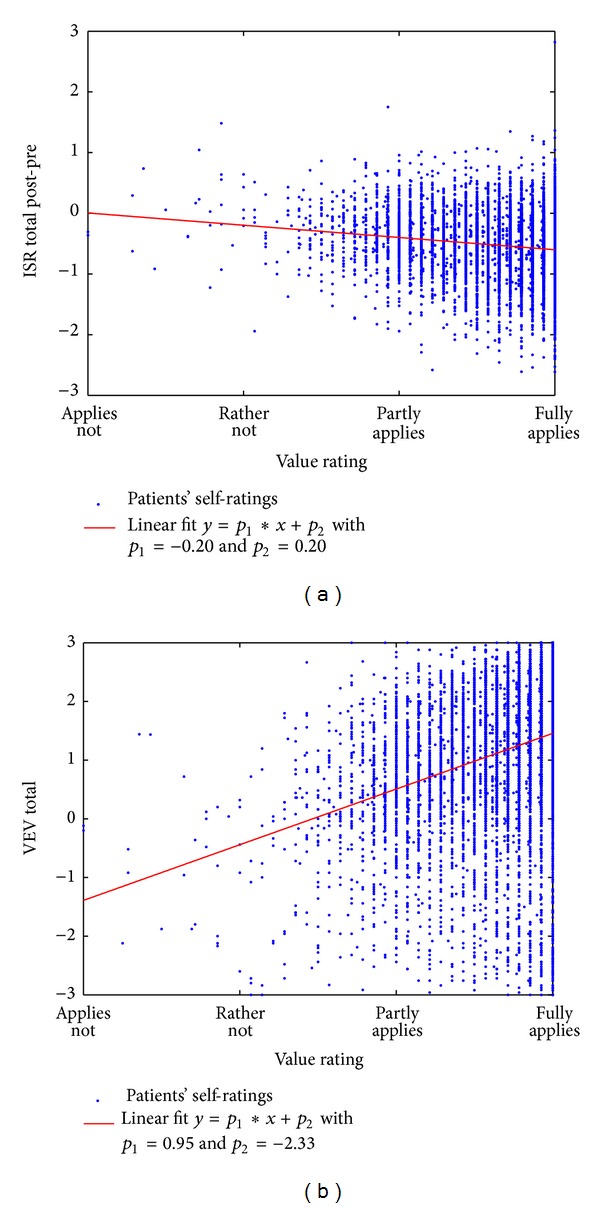
The distribution of the total HF value ratings is visualized in a scatter plot which on the vertical axis represents either the ISR total change (a) or the VEV-K total change (b) after versus before treatment. A linear fit function is inserted to illustrate the dependences of HF value rating and therapeutic effect.

The question how the perception and concordance of the HF values connect the therapeutic effect was answered by a regression analysis with the HF value ratings as independent variable and (a) the total score of the ISR change before and after treatment or (b) the total VEV-K score after treatment as dependent variables. The results are visualized in Figure 3 using scatter plots with linear fit functions. The ISR total change included 7259 patients and showed a highly significant trend in an *F* statistic versus constant model with *F* = 169,  *p* = 3.13*e* − 38. The fit function suggests no symptom reduction for people who did not find the HF values realized in their clinical stay, while the treatment effect developed with the agreement to the HF values.

The VEV-K total score showed an even more prominent dependence with the HF value ratings by offering an *F* value of 807 (*p* = 4.63*e* − 169 with *N* = 7843). Here the fit function suggests a decline of physical and mental health parameters if people do not agree with the HF values, while the same amount of improvement would be achieved in patients who fully find the HF values applied.

As a result of this analysis we are supposing that the HF values might have a salutogenetic effect in the sense that physical and mental health improvement as well as symptom reduction is connected to the subjective experience of the realization of values. Due to the unknown causation and more generally speaking, we can only conclude that those patients who rated themselves as having achieved greater improvements could find the HF values more present in their own personal experience of the environment.

Answering the question “who are the patients who cannot find those values realized in the clinical setting?” already before treatment requires a closer look to the pretreatment survey completed at reception. Here, contentedness ratings as well as the supplementary variables of the ISR significantly differed between “agreeing” and “disagreeing” patients to the realization of the values (*t* = 2.40,  *P* < 0.02 and *t* = 3.45,  *P* < 0.001). Patients who could not or rather not find the values implemented in the clinical setting were less content and had a higher load in symptoms, however, not in depression. Out of 82 “disagreeing” patients 74 (90%) had problems with sleep, 74 (90%) had memory problems, 55 (67%) had problems with their sexuality, 35 (43%) were suicidal, 44 (54%) suffered from recalls of negative memory, and 26 (32%) suffered from PTSD symptoms.

### 3.4. General Treatment Effects (Q3)

**Figure 4:**
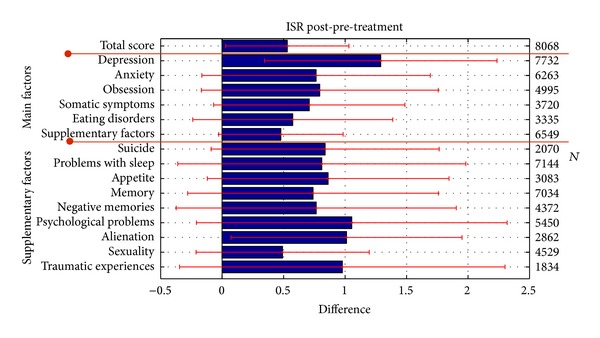
Symptomatic changes after treatment were assessed using the ISR self-rating questionnaire. Here the scale differences were used as a measure with standard error bars.

The treatment effect of a salutogenetic concept can be measured through symptomatic assessments but should also include measures of general health parameters. The improvements of the latter are given by the VEV-K and the GV questionnaire data. The ISR differences between pre- and posttreatment data depict the symptom reductions during treatment. For each factor of the ISR, only those patients were included in the analysis who completed the respective items and who showed a symptomatic load already at reception on this factor. This analysis included patients of all diagnoses. The number of patients included in each factor is listed on the right in each figure.  Table 3 gives the effect sizes corresponding to the mean differences displayed in Figure 4. The total score of the ISR revealed a high effect size of −1.06. Highest symptom reductions were achieved in the depression scale (*d* = −1.37,  *N* = 7732) which also was the major diagnosis. High effect sizes (*d* > 0.8) were also achieved in the reduction of somatic symptoms, anxiety, and obsession. The smallest but still medium effect size in the main factors showed the reduction of eating disorders (*d* = 0.71). It should be noted that due to the high symptomatic load in depression (2.45) in 96% of patients at the beginning of treatment, the main therapeutic effects are intended to the reduction of depression. In contrast, eating disorders were only self-diagnosed with the ISR by 41% of patients with an average load of 1.53.

**Figure 5:**
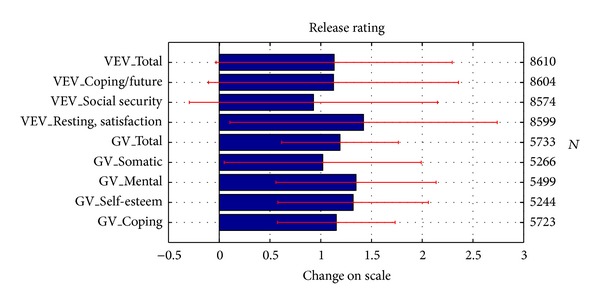
Changes of general health parameters after treatment. Population sizes are indicated on the right vertical axis. The horizontal axis indicates the changes in points on the scale.

The health change questionnaires GV and VEV-K present a quite balanced picture in post-treatment changes across the various factors as can be seen in Figure 5. Most scales are in the range between slight (1) and medium (2) improvement. The ratings of calmness and life satisfaction (VEV-K) actually offered the highest improvements. High improvements also were found in mental health and well-being (GV), self-esteem (GV), and future orientation and coping (VEV-K and GV). All these factors are elements which are in the focus of the therapeutic process and environment in HF indicating a holistic therapy. Finally, the spirituality aspect of transpersonal trust as measured by the TPV showed a medium treatment effect size of 0.69 (*N* = 7215).

### 3.5. Diagnosis-Specific Treatment Effects (Q4)

**Figure 6:**
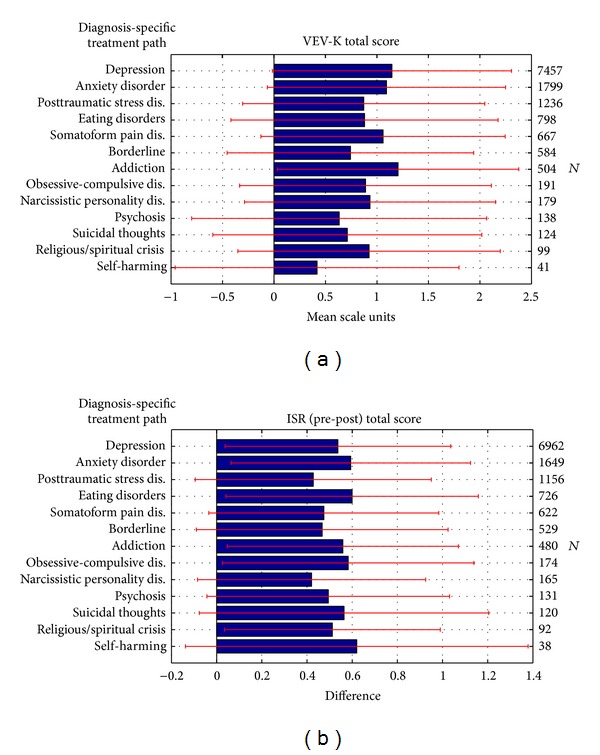
(a) The VEV-K total score changes depending on the treatment path which is related to the main diagnosis. (b) The effect sizes in the improvement of ISR total scores of pre- versus post-treatment in dependence of the treatment path which is related to the main diagnosis. In red, the corresponding standard error bars are depicted.

The question arises how such a treatment concept can deal with the variety of different diagnoses. This question was answered by looking at the symptom and health-related changes of patients' self-ratings who participated in different treatment paths. In Figure 6 the treatment paths are named according to the diagnosis and arranged vertically in the order of the number of patients within a path. Since many patients had several diagnoses, some patients might have changed the treatment path and thus were present in more than one path. The mean changes in total scores of the general health factor of the VEV-K and the overall symptom changes measured by the ISR total score are displayed. The treatment effect sizes are listed in Table 4. The highest VEV-K health changes larger than 1 scale unit are visible for the addiction group, the depression group, the anxiety, and the somatoform pain disorder groups. All differences in overall symptom ratings were between 0.43 and 0.62. In terms of treatment effect sizes the high improvements (*d* > 0.8) were achieved for the paths depression, anxiety disorder, eating disorders, addiction, obsessive-compulsive disorder, psychosis, suicidal thoughts, religious/spiritual crisis, and self-harming. 

The remaining groups PTSD, somatoform pain disorders, borderline, and narcissistic personality disorders revealed medium effect sizes (*d* > 0.6). Patients with these diagnoses, however, suffer from chronic and severe psychological disorders that often have developed over more than 15 years. Such disorders which have developed from childhood on manifest themselves as personality traits (borderline, narcissistic personality disorders). Additionally, there is a high comorbidity in those symptoms that negatively affect the chances for healing. Therefore, besides an inpatient hospital stay additional long-term outpatient psychotherapy is required for treating those disorders. 

### 3.6. Sustainability of Treatment Effects (Q5)

**Figure 7:**
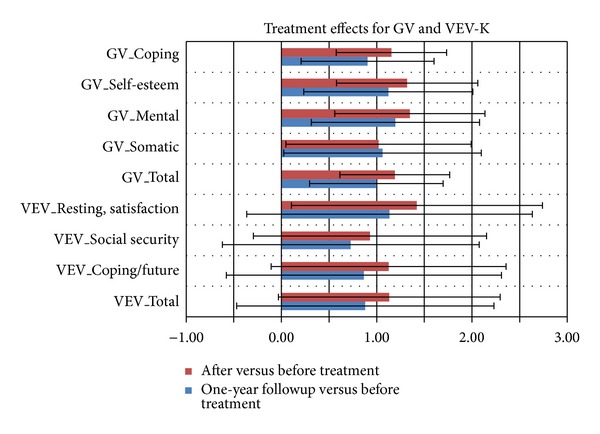
Comparison of changes in general health parameters directly after treatment and in a one-year follow-up survey. Between 950 and 1046 patients are averaged in each bar.

While the term “healing” is often perceived as an act of repairing a dysfunctional human system, the term “salutogenesis” includes the understanding of empowering the patient to generate a state of health. A salutogenetic treatment should additionally provide tools and abilities to a patient to retain and generate health. In this perspective a salutogenetic treatment should present a basis for long-term stability in health-related parameters. In HF, a follow-up survey has been sent out to some patients one year after treatment.  Figure 7 illustrates the long-term changes related to the health change questionnaires and compares the immediate treatment effects at discharge time with the ratings one year after treatment. In those patients who completed the follow-up assessment, changes relative to the pretreatment phase were slightly but not significantly reduced compared to the immediate evaluation at the end of the treatment. Table 4 presents the sustainability of the effect size of the ISR symptom reduction. Here, patients with depression, anxiety disorders, and PTSD retain their symptom reduction to 82, 88, and 92%. The high numbers after release in patients suffering from addiction and obsessive-compulsive disorders even increase after one year by 16 and 9%. The largest reduction of symptom improvement (down to about 50%) showed patients suffering from psychosis, suicidal thoughts, and self-harming. Those patients might additionally need an individual psychotherapy rather than group therapy only. At average 79% of symptom, improvement is maintained after one year. Patients treated in the paths of psychotic disorders, suicidal thoughts, and self-harming are those with the most severe symptoms and ICD10 diagnoses. This is especially true in those forms of PTSD caused in early childhood and requires long-term psychotherapy. An inpatient hospital stay of ten weeks, however, may only reduce acute symptoms associated with psychotic experiences, suicidal attempts, and self-harming behavior. Patients with traumatic experiences in early childhood are often unable to maintain interpersonal relationships. Those patients profit from the community and group therapy during the hospital stay but are unable to maintain the social relations in their daily life. In our opinion only a consistent (parental-like) subsequent “nutrition” with the early human needs for security and confidence could improve such patients' health state. However, to date no catamnestic data across 5 or 10 years are available.

The connection between symptom reduction and the improvement in health-related factors of a wider concept of health as assessed by the VEV-K and GV was exemplarily studied in patients who participated in the depression path. Changes on the depression scale of the ISR were correlated with the factors of the VEV-K and GV using Spearman's rank correlations. As shown in Table 5, all 4 VEV-K factors and all 5 GV factors exhibited moderate correlations with *r* between −0.33 and −0.45. Correlations larger than −0.40 were found for GV_Total, the coping aspect of the GV, and the GV mental health improvement. The VEV-K factor “coping and future orientation” offered an *r* = −0.38. This confirms the hypothesis that acquisition of coping abilities and strategies seems to be an important factor in the treatment of depressive disorders as well as the improvement in mental health. Assessing those correlations one year after treatment, we still found an improvement in coping (*r* = −0.47) connected with the sustainability of the treatment effect, which indicates that some patients actually seem to have gained skills for coping, self-management, and future orientation which they could successfully apply in their daily lives afterwards.

### 3.7. Comparison of Treatment Effects with Other Clinics (Q6)

The above-reported results can be set in relation to the treatment effects of 14 other German clinics. 

Table 6 shows a selection of comparable data extracted from the IQP report using Psy-BaDo. There is a significant difference between the mean improvement in ISR as well as most GV scores. Most remarkable are improvements of 1.35 in HF and 1.18 in the other clinics on the ISR depression scale. The personal change due to treatment as assessed by the VEV-K was large in both groups. The GV results demonstrate that, on average, patients found essential improvement in different aspects of life. Factors such as psychological health (HF median = 4.69), self-esteem (HF median = 4.68), and well-being (HF median = 4.68) show good and significantly greater improvements than the other clinics. Only the disease comprehension scale was significantly higher in the other clinics (median = 5.2 versus HF median 4.88).

## 4. Conclusion

Starting with the assumption that a sustainable form of healing in a psychosomatic inpatient clinic requires more than the focus on symptom reduction, we presented one approach of a psychosomatic hospital in Germany that follows a holistic and specific salutogenetic approach. It should be noted that several German psychosomatic clinics follow similar salutogenetic ideas to a certain extend. The approach of the HF clinics was formulated in a guideline that includes a set of values intended to generate salutogenetic behavior and experience within the whole clinic environment. Also, patients are supported in living a health-oriented and self-directed life. 

In the first part of this study our aim is to demonstrate whether and how several aspects of human values underlying the holistic concept have been implemented in a clinical setting for inpatient treatment of psychosomatic disorders. Therefore, the clinic has developed a self-assessment questionnaire. The results suggest that salutogenetic values have not only been formulated in the clinical guidelines but were also successfully transferred to the therapeutic environment. This self-assessment shows that 90 to 99% of the patients agreed to the statement that those values were realized and present in the clinic. Unfortunately, the high concordance with the questionnaire statements moved the distribution towards the upper end of the scale and created a saturation effect with a non-Gaussian distribution. Therefore, we did not perform correlational analysis with treatment effects. Nevertheless, the high number of patients allowed a linear model to be fitted to investigate the connection between patients' perception of the clinical value codex and the therapeutic effect. The strong dependency between symptom reduction and value ratings and the total score of health changes (VEV-K) suggests that it is important for a hospital to offer a supportive environment that allows patients to get involved with salutogenetic factors. Those factors were intended to be realized not only within therapeutic sessions but also in other areas of the patients' daily life in the clinic. The statements about concordance with the ethical values may be biased, due to patients wanting to express their gratitude towards the clinic. We took a closer look at those patients with low concordance ratings, and found that they fit a certain profile. They were less content and offered a higher load in symptoms, however, not in depression. From 56 “disagreeing” patients, 89% had sleeping problems, 88% memory problems, 70% had problems with their sexuality, 36% were suicidal, and 32% suffered from PTSD symptoms.

Data acquisition for measuring the therapeutic outcome was performed with self-assessment inventories as well. We are aware of the short comings of self-assessment questionnaires but want to point out that, particularly in the context of psychosomatic disorders, perceived improvements in health are very valuable to the individual, regardless of the explanations for these changes such as possible placebo effects or insufficiencies of the questionnaires. It should be noted that an F diagnosis assessed by a therapist also is based on patients' reports. Therefore, it is not a surprise that, according to the IQP, the self-assessments are roughly in line with the diagnoses given by the therapist. For some measures, especially those asking for mental states and subjective experiences, one has to rely on the self-ratings for now. Nonetheless, the analyses would benefit from an inclusion of more popular questionnaires such as the SCL90. However, the clinic intends to use questionnaires with fewer items and prefers license-free inventories. Further, it might be interesting to additionally measure the HF values in other clinics to find out whether the ceiling effects occur there as well and for a better understanding and possible confirmation of the importance of these factors.

We have introduced salutogenesis as a complex construct with many different aspects. Salutogenetic factors are inherent in concepts such as mindfulness, spirituality, social support, and the sense of coherence that comprises comprehensibility, manageability, and meaningfulness. Aspects of those concepts were found to a certain extend in the HF guidelines and/or the treatment concept of the clinic. However, despite not all of the salutogenetic factors were explicitly assessed in the questionnaires, the questionnaires VEV-K, GV, and the TPV used in HF during several years cover those aspects to a certain extent.

The sustainability of improvements in a 1-year follow-up was about 80% and can be regarded as high. However, these data are only available for a small proportion of total patients. Comparing the posttreatment data of the follow-up group with the posttreatment group of all patients, those patients who returned the follow-up questionnaire already showed higher treatment effect sizes at release time. Therefore, it is questionable whether patients with lower treatment success—often those with more severe symptoms—would also display such high sustainability. The fact that treatment paths involving patients with more severe symptoms showed a far lower sustainability argues against it. 

In direct comparison between HF and the other clinics it should be noted that while the distribution in age and gender was comparable, the number of patients with a private health insurance was 70% in HF but less than 10% in other clinics. This also links to a significantly higher education level in HF compared to other clinics. Furthermore, the average treatment duration was about 14 days longer in HF than in other clinics (57 days versus 43 days). Since both parameters, educational level and treatment time, are positively related to health improvement, the superiority of the HF concept over the concept of other clinics cannot be definitely stated and would require further analysis. Considering all results, the data suggest that the perceived successful implementation of a guideline that is based on the idea of salutogenesis affects patients' health state in a positive way. However, as other clinics also follow more or less salutogenetic approaches, the comparison between clinics is not very meaningful.

Successful implementation of a salutogenetic concept requires, through the entire hospital staff, the promotion of an autonomous development and dialog of values, including meaningfulness and spirituality. Patients might then profit from the inner attitude they perceive from everybody within the clinical environment, possibly enabling them to integrate this experience as an ideal for their own coping strategies leading to an increase of their SOC. An interesting future study could be to explore the effect of the inner attitude of the therapist on the treatment success of their patients.

The highest symptom reductions were achieved in depression, which was also most commonly diagnosed and also was the most frequent treatment path. A correlation analysis of patients with depression revealed medium but sustained correlations between the improvements in coping-related aspects and depressive symptoms. This supports the idea that proactive behavior (manageability in the SOC model) is a key feature for health.

The TPV measures different facets of religiosity related to a transpersonal trust. The pre-post-treatment comparison only shows medium effect sizes. Probably, the gain in transpersonal trust seems not to be as strongly promoted in the clinic as other forms of spirituality. This assumption can be derived from the observation that 90% of the patients agree that HF is partly or fully a place of spiritual growth. To address this issue, additional tests investigating meaning and spirituality, for example, LEBE by Schnell and Becker [64], would be useful. In contrast, the VEV.Change_Resting factor that can be regarded as a scale related to the concept of mindfulness offered a high relationship with health improvement. Therefore, distinguishing between religious and spiritual coping aspects seems to be important. It may be useful to further analyze the effects of mindfulness in psychosomatic treatment with respect to the various ICD10 diagnoses. Unfortunately, the measurement of the aspect of mindfulness was not explicitly determined. It could be suggested to use the Freiburg Mindfulness Inventory (FMI) or another accepted mindfulness inventory at least with a group of patients.

With these findings we would like to propose the idea that a salutogenetic treatment requires a value driven social environment that provides a learning atmosphere for the acquisition of health supporting skills, behavior, and attitudes and finally leads the body and mind into a state of self-recovery and to the development of skills for further “health development” in order to continue the recovery process in daily life. We therefore propose that salutogenesis should be initiated in the clinical environment and in the therapeutic processes, with a continuation beyond the clinical stay. This goal can only be met when patients learn in the clinic setting how to live a health promoting, self-directed, and fulfilled life. 

## Supplementary Material

The Supplementary Material comprises a comprehensive list of questionnaires and items used for the analysis in English translation. These are the ISR, TPV-10, GV, VEV-K and HF-values.

## Figures and Tables

**Table 1:** The data sets and its underlying patient population are listed for each research question.

Research question	Inventory/data set	Subgroups	Number of patients
Q1	HFV total and 14 items	All patients*	7987 to 8170

Q2	HFV total (i.v.) ISR total effect size (d.v.) VEV-K total change (d.v.)	All patients*	7259 7843

Q3	ISR (pre + post) (total + subscales) VEV-K (total + 3 subscales) GV (total and 4 subscales)	All patients*	Up to 8068

Q4	ISR total effect size VEV-K total change	Patients in 13 paths*	38 to 6962 41 to 7457

Q5	ISR pre + follow up VEV-K (total + 3 subscales) followup GV (total and 4 subscales) followup	Follow-up return*	Up to 1046

Q6	ISR (pre + post) (total + subscales) VEV-K (total) GV (all 11 items)	Patients in 2011	1181 (HF) 5348 (other clinics)

*As many patients as possible were included who completed the particular questionnaire scale partly or fully. i.v.: independent variable; d.v.: dependent variable.

**Table 2:** The mean rating is the average of between 7987 and 8170 patients on a scale between ^1^not applies and ^4^fully applies. SD is the corresponding standard deviation. The ratio evaluation columns show the percentage of patients who voted with “fully applies” (left) or with “partly or fully applies” (right).

Heiligenfeld values	Mean rating	Ratings in % of patients			
		Fully applies^4^	Partly applies^3^	Rather not applies^2^	Applies not^1^
Heiligenfeld is a…					
human place	3.84	84.6	14.5	0.6	0.3
healthy place	3.63	65.9	31.1	2.6	0.4
place of healing	3.66	68.5	28.7	2.5	0.3
place of love	3.43	50.4	42.7	5.9	0.9
place of wholeness	3.48	55.9	37.0	6.1	0.9
place of spiritual growth	3.42	54.0	35.9	7.8	2.3
place of community	3.78	79.1	19.1	1.5	0.3
place of mindfulness	3.49	54.4	40.4	4.8	0.5
I think, in Heiligenfeld…					
fundamental rights are respected	3.77	78.0	20.6	1.2	0.2
people are being acknowledged and appreciated	3.74	76.4	21.3	1.9	0.3
the essential of life is touched	3.68	71.1	25.7	2.9	0.3
responsibility is experienced	3.54	59.4	35.9	4.2	0.5
life is respected	3.81	81.8	16.9	1.2	0.1
empathy is experienced	3.73	75.7	22.1	2.0	0.2

Mean of all items	3.64	91.3	8.3	0.3	0.0

**Table 3:** Symptom-related treatment effects. Effect sizes (Cohen's *d*) with respective population size are indicated for the post-pre-treatment.

ISR total ISR subfactors TPV total	Pre-prost-treatment	
	Effect size (Cohen's *d*)	*N *
Total score	1.06	8068
Depression	1.37	7732
Anxiety	0.82	6263
Obsession	0.82	4995
Somatic symptoms	0.91	3720
Eating disorders	0.71	3335
All supplementary factors	0.95	6549
Suicide	0.90	2070
Problems with sleep	0.69	7144
Appetite	0.88	3083
Memory	0.73	7034
Negative memories	0.67	4372
Psychological problems	0.83	5450
Alienation	1.08	2862
Sexuality	0.70	4529
Traumatic experiences	0.74	1834
TPV total	0.69	7215

**Table 4:** Total symptom rating for each of the 13 treatment paths. Effect sizes (Cohen's *d*) with respective population size are indicated for the post-pretreatment as well as the follow-up-pretreatment comparisons.

Diagnosis-specific treatment path	ISR total scores					
	Post-pre-treatment				Follow-up pretreatment	
	Effect size *d *	*N *	Effect size *d *	*N *	Effect size *d *	*N *
Depression	0.91	6962	1.07	677	0.87	677
Anxiety disorder	0.98	1649	1.13	178	1.00	178
PTSD	0.64	1156	0.82	109	0.76	109
Eating disorders	0.95	726	0.93	76	0.70	76
Somatoform pain disorders	0.76	622	0.77	69	0.63	69
Borderline	0.71	529	0.81	71	0.69	71
Addiction	0.97	480	0.83	43	0.96	43
Obsessive-compulsive dis.	0.95	174	0.78	23	0.85	23
Narcissistic personality dis.	0.60	165	0.81	29	0.56	29
Psychosis	0.80	131	1.17	13	0.60	13
Suicidal thoughts	0.86	120	1.62	9	0.82	9
Religious/spiritual crisis	0.82	92	1.13	12	0.91	12
Self-harming	0.92	38	0.51	2	0.24	2

**Table 5:** Correlational analysis between depression symptom changes of patients with depression as main diagnosis and health change factors of the VEV-K and GV. Post-pre-treatment: *N* > 5000, all *P* values <1*e* − 10.

Health change factor (that was correlated with the ISR depression scale)	Spearman's *r* Post-pre-treatment	Spearman's *r* follow-up pretreatment
VEV_Total	−0.39	−0.36
VEV_Coping/future	−0.38	−0.37
VEV_Social security	−0.33	−0.28
VEV_Resting/satisfaction	−0.35	−0.31
GV_Total	**−0.45**	**−0.51**
GV_Somatic	−0.33	−0.39
GV_Mental	**−0.42**	**−0.50**
GV_Self-esteem	−0.33	**−0.42**
GV_Coping	**−0.41**	**−0.47**

Post-pre-treatment: *N* > 5000, all *P* values <1*e* − 10. Follow-up pretreatment: *N* > 600, all *P* values <1*e* − 10.

Bold values indicate correlations larger than 0.4.

**Table 6:** Psychosomatic treatment effects in comparison between HF and 14 other German clinics. A paired *t*-test was performed on pretreatment and posttreatment data. The Pearson *χ* ^2^-test was applied to the post-treatment data.

Scale	HF clinic mean (SD)	Other 14 clinics mean (SD)	Significance test
ISR total pre-post	0.58 (±0.48)	0.52 (±0.51)	*t* = 3.84, *P* < .001
ISR depression pre-post	1.35 (±0.97)	1.18 (±0.96)	*t* = 10.31, *P* < .001
VEV-K	129.3 (±26.9)	128.3 (±26.7)	*t* = −1.03, n.s.
GV physical	4.42 (±0.73)	4.22 (±0.77)	*χ* ^2^ = 45.8, *P* < .001
GV psychological	4.69 (±0.87)	4.44 (±1)	*χ* ^2^ = 80.3, *P* < .001
GV self-esteem	4.68 (±0.83)	4.38 (±0.92)	*χ* ^2^ = 103.4, *P* < .001
GV social problems	4.07 (±0.88)	4.05 (±0.94)	*χ* ^2^ = 7.8, n.s.
GV private relationships	4.4 (±0.83)	4.26 (±0.95)	*χ* ^2^ = 47.0, *P* < .001
GV occupational	3.89 (±0.83)	3.77 (±0.92)	*χ* ^2^ = 50.1, *P* < .001
GV motivation	4.39 (±0.79)	4.32 (±0.88)	*χ* ^2^ = 50.6, *P* < .001
GV comprehension	4.88 (±0.84)	5.2 (±1.13)	*χ* ^2^ = 69.5, *P* < .001
GV future orientation	4.51 (±0.9)	4.34 (±1.02)	*χ* ^2^ = 49.7, *P* < .001
GV well-being	4.68 (±0.85)	4.4 (±1.02)	*χ* ^2^ = 94.6, *P* < .001
GV daily life requirements	4.33 (±0.81)	4.19 (±0.88)	*χ* ^2^ = 31.7, *P* < .001
