# Supplementary material for: Evaluation of a Salutogenetic Concept for Inpatient Psychosomatic Treatment
Source: Evid Based Complement Alternat Med. 2013 Sep 16;2013:735731. doi: 10.1155/2013/735731 (PMC3789398; doi:10.1155/2013/735731)
Supplement: Supplementary file 1 — The Supplementary Material comprises a comprehensive list of questionnaires and items used for the analysis in English translation. These are the ISR, TPV-10, GV, VEV-K and HF-values. [file 735731.f1.docx]

# Appendix

## Questionnaire surveys

The following comprehensive list of questionnaires had to be completed at reception and/or release by all patients in the Heiligenfeld Clinics Bad Kissingen, Germany. All questionnaires were in German and were translated into English for this publication.

**Questionnaires to be completed at reception, release and after one year follow-up:**

### ISR (ICD-10 Symptom rating)

A list of the ISR symptom rating factors is given here:

1. depression
2. anxiety
3. obsession
4. somatic symptoms
5. eating disorders
6. supplementary factors
   1. suicide
   2. problems with sleep
   3. memory
   4. sexuality
   5. traumatic experiences

### TPV-10 (Transpersonal confidence questionnaire)

Please mark with a cross in which degree the following statements apply to you in your current situation. In case of doubt choose the answer which most likely applies to you. Answer briskly and please keep in mind to answer all of the questions although they might be similar.

**Scale: Fully applies, partially applies, does rather not apply, does not apply at all**

1. I feel connected to a higher reality/ a higher being/ god. I can rely on he/she/it in difficult times
2. I am part of a greater whole, in which I was born
3. I am a human being with a body and an intellect. And I am also inseparable linked to the cosmos
4. We humans cannot determine everything. There is a higher reality/ a higher being/ god which I can trust in
5. Sometimes I have the feeling that in my life, I am led by a higher understanding of things
6. I characterize myself as being religious, although I do not belong to any denomination
7. Religious practices (e.g. praying, chanting mantras, singing holy songs, meditating) help me in difficult situations
8. I try to place my trust in god/ a higher being
9. My sole lives on posthumously
10. I have already made the experience to feel at one with the world and the cosmos

## Questionnaires to be completed at release and follow-up:

### GV (Global changes in mental and physical states)

In your assessment, please consider your time shortly before your treatment here until now

**Scale: Considerably improved, partially improved, no change, partially declined, considerably declined, cannot be assessed, was not my issue**

1. How has the disorder of your personal well-being (physical symptoms) changed?
2. How has your mental- health problem (psychological symptoms) changed?
3. How has your self- acceptance/your self-esteem changed? Do you like yourself more or rather less now?
4. How have your social problems (e.g. employment problems, housing problems, financial problems) changed?
5. How have your relationships to people who are very important to you changed, considering your private area (parents, partner, children, friends etc.)?
6. How has your attitude or your relationship changed towards people who are very important to you, considering the job area (boss, colleagues)?
7. How has your capability for own initiative and taking responsibility for your life (e.g. the ability to establish contact, assertiveness) changed?
8. How has your understanding of illness changed? Do you feel the possibility to change your life through your own actions as you now understand what caused your problems in the first place?
9. How has your attitude changed towards your future (considering achievable future plans)?
10. How has your emotional well-being changed?
11. How has your assessment towards your ability to fulfill daily demands changed?

**Scale: Very satisfied, partially satisfied, undecided, not really satisfied, not satisfied at all**

1. Considering the result of my therapy in Heiligenfeld, I feel

### VEV-K (Comparative questionnaire of behavior and experience)

Pleas mark with a cross, to what extend you notice the changes. Please only one cross per row.

**Compared to the time immediately before therapy in Heiligenfeld I…**

**Scales: True, not true Strong, medium, slightly, neither…nor**

1. I feel less harried
2. I am more relaxed
3. I do not get worked up over a lot of things anymore
4. I feel calmly and balanced
5. I am at peace within
6. I face difficulties more calmly
7. I am not afraid anymore to fail at something I should succeed in
8. I take unexpected events more calmly
9. I now know better what I want to do and what I can do
10. I see a clear picture of me and my future
11. I feel less worried thinking of my future
12. I think I now know better what is important to me
13. I am somehow happy and more optimistic
14. I am more cheerful
15. I cannot fully grasp it yet, but I feel better for some time past
16. I somehow see more sense in my life
17. I feel less dependent on others
18. I feel more satisfied with and less worried about myself
19. I am less at war with myself
20. I can better accept my problems
21. I do not suffer from my own insecurity
22. everything bears a meaning and is alive
23. I became more relaxed dealing with other people
24. I can now express myself more freely
25. I do not feel uneasy anymore talking to others

In what other areas have you changed?

### Heiligenfeld value questionnaire

Consider the clinic under the impression on you and the impact on you as a whole. Only one cross per row.

**I experience Heiligenfeld as a…**

1. human place
2. healthy place
3. place of healing
4. place of love
5. place of wholeness
6. place of spiritual growth
7. place of community
8. place of mindfulness

**I think in Heiligenfeld…**

1. fundamental rights are respected
2. people are being acknowledged and appreciated
3. the essential of life is touched
4. responsibility is experienced
5. life is respected
6. empathy is experienced
